# The role of psychosocial factors in patients’ recollections of breast reconstruction options discussed with their surgeons

**DOI:** 10.1038/s41598-022-11478-0

**Published:** 2022-05-06

**Authors:** Haoqi Wang, Jun Liu, Mary Catherine Bordes, Deepti Chopra, Gregory P. Reece, Mia K. Markey, Aubri S. Hoffman

**Affiliations:** 1 Department of Biomedical Engineering, The University of Texas at Austin, Austin, TX USA; 2 Department of Plastic Surgery, The University of Texas MD Anderson Cancer Center, Houston, TX USA; 3 Department of Psychiatry, The University of Texas MD Anderson Cancer Center, Houston, TX USA; 4 Department of Imaging Physics, The University of Texas MD Anderson Cancer Center, Houston, TX USA; 5 The Value Institute for Health and Care at Dell Medical School, The University of Texas at Austin, 1601 Trinity St. Bldg. B, StopZ1600, Austin, TX 78712 USA

**Keywords:** Cancer, Psychology, Health care

## Abstract

A patient’s comprehension and memory of conversations with their providers plays an important role in their healthcare. Adult breast cancer patients whose legal sex was female and who underwent treatment at the Center for Reconstructive Surgery at The University of Texas MD Anderson Cancer Center were asked to indicate which breast reconstruction procedures they discussed with their surgeon. We focused on the three most frequent responses: (a) participants who remembered discussing implant-based, tissue-based, and combination procedures; (b) participants who remembered only an implant-based option being discussed; and (c) participants who remember only a tissue-based option being discussed. We used multinomial logistic regression models to explore the psychosocial factors associated with patients’ recollections of their breast reconstruction options after discussions with their reconstructive surgeons, controlling for medical factors that impact surgical decision-making. Our analyses identified body mass index, body image investment, and body image as statistically significantly associated with the reconstructive options that a participant recalls discussing with their surgeon. Our findings highlight body image investment and body image as important psychological factors that may influence what patients remember from consultations about breast reconstruction options.

## Introduction

Informed consent^1,2^ and shared decision-making^3^ require that patients understand and remember information provided by their healthcare providers about their condition, treatment options, and risks. Patients' ability to recall information from conversations with their providers predicts their satisfaction with care and adherence to healthcare advice^4–6^. However, patients may struggle to understand and recall information, particularly when processing life-threatening news or envisioning future outcomes^7,8^. Previous studies indicate that the more information that is given, the smaller the proportion of the conversation that is correctly remembered^9^, and that 40–80% is forgotten immediately^10,11^. In some scenarios, it is estimated that almost half of the information that is remembered is incorrect^12^.

Many medical communication models have been proposed to analyze comprehension and recall of medical consultations^13,14^. According to the classic transactional model of communication^15^, there are three key components to a conversation between a provider and patient. First, there are factors related to the provider, such as the use of specialized medical terminology^16^ and the amount and order of information presented^17^. Second, the media of information presentation must be considered. While verbal communication is faster, written information is often recalled better^18^. Pictures linked to text can increase recall of health education information, compared with text alone^19^. The third component of the transactional model of communication is factors related to the patient. Both age^20,21^ and neoadjuvant chemotherapy^22^ can diminish recollection, whereas higher health literacy is associated with greater comprehension and memory of conversations^23^. It is also critical that the patient actively participate in the conversation since people remember what they said more than what the other person said^24^. Finally, the patient’s emotional state can significantly impact understanding and recall of information. Patients under stress, for example because of an upcoming surgical procedure^25^, experience attentional narrowing such that important information may not be stored^26,27^. Unusually high and unusually low anxiety levels are also associated with impaired recall^28^.

The purpose of this study was to explore the psychosocial factors associated with cancer patients’ recollections of their breast reconstruction options after discussions with their reconstructive surgeons, controlling for medical factors that impact surgical decision-making. Breast reconstructive options for an individual patient depend on both surgical factors and medical factors such as body mass index (BMI) and age. For example, higher BMI is associated with higher rates of morbidity in tissue-based reconstruction^29^ and older patients are at higher risk of postoperative complications^30^. On the other hand, psychological factors, such as body image investment (i.e., the degree to which someone attends to their body’s appearance as a component of their self-worth), do not change a patient’s eligibility for different reconstruction options. Nevertheless, such psychological factors could influence what a patient *remembers* about their options for reconstruction. For women with breast cancer, it is important to understand their emotional investment in the shape of their breasts (for example, as part of their perceived feminine identity or sexuality) and the impact that investment may have on recalling the options available to them. We hypothesized that a patient's subjective experience of their body is associated with which breast reconstruction options they remember.

## Methods

### Study sample

We analyzed data from a prospective Institutional Review Board-approved research project (IRB #2010-0321) that enrolled patients at various stages of breast reconstruction. The study was approved by the Institutional Review Board of The University of Texas MD Anderson Cancer Center. All methods were carried out in accordance with relevant guidelines and regulations. The current study was not part of the original study and there was no prospective data collection component to the current study. The study sample consisted of adult breast cancer patients whose legal sex was female and who underwent treatment at the Center for Reconstructive Surgery at The University of Texas MD Anderson Cancer Center from 2011 to 2014. Participants provided written informed consent. We used data collected from 505 participants’ baseline visits.

At the baseline study visit, participants were asked to indicate which reconstruction procedures they discussed with their surgeon (choose all that apply): (1) implant-based (i.e., tissue expander and implant), (2) tissue-based (i.e., transverse rectus abdominis myocutaneous, deep inferior epigastric perforator, or superficial inferior epigastric artery flaps), and (3) combination of implant- and tissue-based (i.e., latissimus dorsi reconstruction with tissue expander and implant). There are eight possible answers to this question, but some were reported by only a few participants, so we focused on the three most frequent responses: (a) participants who remembered discussing all three reconstruction categories, i.e., checked the boxes for implant-based, tissue-based, and combination; (b) participants who remembered only an implant-based option being discussed, i.e., checked the implant-based box only; and (c) participants who remember only a tissue-based option being discussed, i.e., checked the tissue-based box only. There were 306 participants included in the sample after filtering for these three groups.

### Demographic characteristics

Participant race and ethnicity were obtained via self-report questionnaires. Participants’ age, height, and weight were abstracted from their medical record.

### Body image

The Body Image Scale^31^ (BIS) is a 10-item self-report measure of body image dissatisfaction used to assess body image changes in patients with cancer. Participants rated their dissatisfaction on a 4-point scale from 0 (not at all) to 3 (very much). The overall score ranges from 0 to 30, with a higher score indicating more body image disturbance. This scale has been reported to have high internal consistency and good validity and is suitable for use in clinical trials^31^.

### Body image investment

The Appearance Schemas Inventory-Revised^32–34^ (ASI-R) is a 20-item measure that assesses body image investment; in other words, it assesses beliefs or assumptions about the importance, meaning, and influence of appearance in one's life. It is an important construct in the evaluation of body image concerns. Items were rated on a 5-point Likert scale from 1 ‘strongly disagree’ to 5 ‘strongly agree’. The overall ASI-R score is calculated by averaging the individual item scores. The overall ASI-R score ranges from 1 to 5, where a higher score indicates more body image investment. The instrument and subscales have been reported to have high internal consistency and good validity for breast cancer patients undergoing reconstruction^34^.

### Psychological distress

The Brief Symptom Inventory-18 (BSI-18)^35,36^ is an 18-item screening questionnaire used to assess adult self-reported psychological problems for three dimensions (depression, anxiety and somatization). Participants rated the severity of their symptoms on a 5-point scale from 0 (not at all) to 4 (extremely). The global severity index (GSI) is a total score of all three dimensions of the BSI-18 that indicates psychological distress. The overall GSI score ranges from 0 to 72, with a higher score indicating more distress. This scale has been reported to have high internal consistency to reliably assess psychological distress^37^.

### Statistical methods

Descriptive statistics, including means, standard deviations, medians, and interquartile ranges were used to summarize cardinal and ordinal variables. Frequencies and percentages were used to summarize the categorical clinical characteristics. The Shapiro–Wilk test was used to test normality. If any variable was found to be non-normal, the Kruskal–Wallis test^38^ was used to compare ordinal measures among the three groups. Dunn’s test^39^ was utilized to adjust multiple comparisons. The significance level was adjusted for multiple tests using Holm's correction^40^.

### Univariate and multiple multinomial logistic regression models

Univariate multinomial logistic regression models were performed first. The variables studied were BMI, ASI-R composite score, BIS score, and GSI (from BSI-18). Covariates with a Type III *p*-value of less than 0.2 in the univariate analysis were considered potential predictive factors for the reconstruction options that the patient remembered discussing with their surgeon. A backward model selection approach based on the Akaike information criterion was applied to fit the multivariable multinomial logistic regression model.

Odds ratios were calculated and all tests were two-sided with a *p*-value < 0.05 considered statistically significant. Analyses were performed using R 4.0.5 (R Core Team, 2021)^41^.

## Results

### Sample characteristics

The characteristics of the patient sample are presented in Table 1. Three hundred and six participants were included in the analyses. Their mean age was 49.4 years (SD = 9.6 years) and mean BMI was 28.0 kg/m^2^ (SD = 5.3 kg/m^2^). The majority of participants were White (74.8%) and non-Hispanic (75.2%).

**Table 1:** Patient characteristics.

	Memory group			
	Overall	All options	Tissue-based options	Implant-based options
N (%)	306	50 (16.3)	118 (38.6)	138 (45.1)
Age in years, mean ± SD	49.4 ± 9.6	50.4 ± 11.7	49.5 ± 8.0	48.9 ± 10
BMI in kg/m^2^, median (IQR)	27.10 (7.65)	26.35 (6.70)	29.95 (6.28)	24.90 (5.85)
**Race, N (%)**				
Caucasian	229 (74.8)	43	85	101
African American	29 (9.5)	4	11	14
Asian	14 (4.6)	1	5	8
Other	12 (3.9)	1	5	6
Declined to answer	22 (7.2)	1	12	9
**Ethnicity, N (%)**				
Non-Hispanic	230 (75.2)	42	80	108
Hispanic	57 (18.6)	6	35	16
Not available	19 (6.2)	2	3	14

### Descriptive statistics

Based on the distributions of responses, we analyzed the three primary groups: (a) participants who remembered discussing all three reconstruction categories, i.e., checked the boxes for implant-based, tissue-based, and combination; (b) participants who remembered only an implant-based option being discussed, i.e., checked the implant-based box only; and (c) participants who remember only a tissue-based option being discussed, i.e., checked the tissue-based box only. Fifty (16.3%) participants remembered all three categories of reconstructive procedures being discussed, 138 (45.1%) participants remembered only an implant-based option being discussed, and 118 (38.6%) participants remembered only a tissue-based option being discussed. The median age was 49.5 (IQR = 20) years for participants who remembered discussing all reconstructive categories; 49.0 (IQR = 14.5) years for participants who remembered discussing only an implant-based option; and 50.5 (IQR = 10.75) years for participants who remembered discussing only a tissue-based option.

BMI was statistically significantly different between the three groups. Participants who remembered discussing all reconstructive categories had a median BMI of 26.35 (IQR = 6.70); participants who remembered discussing only an implant-based option had a median BMI of 24.90 (IQR = 5.85); and participants who remembered discussing only a tissue-based option had a median BMI of 29.95 (IQR = 6.28) (Kruskal–Wallis test, *p* < 0.001). Pairwise comparisons showed that BMI was statistically significantly higher for participants who remembered tissue-based options than for those who remembered implant-based options (Dunn’s test, p-adjust < 0.001) or all options (p-adjust < 0.001), which indicates that BMI was associated with which options the patients remembered discussing with their surgeons. Participants with a higher BMI tended to remember discussing only a tissue-based option, whereas participants with a lower BMI tended to remember discussing only an implant-based option.

The BIS^31^ score, which measures body image, differed significantly between the three groups: participants who remembered discussing all reconstructive categories, 5.5 (IQR = 9.25) vs. participants who remembered discussing only an implant-based option, 6.0 (IQR = 10.00) vs. participants who remembered discussing only a tissue-based option, 11.0 (IQR = 11.00) (Kruskal–Wallis test, *p* = 0.002). Pairwise comparisons showed that the BIS score was statistically significantly higher for participants who remembered discussing only a tissue-based option than for participants who remembered discussing only an implant-based option (p-adjust = 0.005) and participants who remembered discussing all reconstructive categories (p-adjust = 0.015).

Scores for the ASI-R^32–34^, which measures body image investment, differed significantly between the three groups: participants who remembered discussing all reconstructive categories, 3.11 ± 0.66 vs. participants who remembered discussing only an implant-based option, 3.32 ± 0.56 vs. participants who remembered discussing only a tissue-based option, 3.19 ± 0.58 (analysis of variance, *p* = 0.046). The Shapiro–Wilk test indicated normal distribution for ASI-R (*p* = 0.451). Tukey multiple comparisons showed that the ASI-R score was marginally higher for participants who remembered discussing only an implant-based option than for participants who remembered discussing all reconstructive categories (p-adjust = 0.073) and participants who remembered discussing only a tissue-based option (p-adjust = 0.150). This indicates that participants with higher body image investment tended to remember discussing only an implant-based option.

The BSI-18^35,36^, which measures psychological distress summarized by the GSI, differed marginally between the three groups: participants who remembered discussing all reconstructive categories, 4 (IQR = 7.75) vs. participants who remembered discussing only an implant-based option, 5 (IQR = 8.00) vs. participants who remembered discussing only a tissue-based option, 6 (IQR = 10.75) (Kruskal–Wallis test, *p* = 0.119). Pairwise comparisons showed that the global severity index (from BSI-18) was marginally higher for participants who remembered discussing only a tissue-based option than for participants who remembered discussing all reconstructive categories (p-adjust = 0.140), i.e., participants who remembered discussing only a tissue-based option were more distressed than participants who remembered discussing all reconstructive categories, although this difference was not statistically significant.

### Univariate analysis

We investigated medical factors (BMI, age), general psychological distress (global severity index from BSI-18), body image investment (ASI-R), and body image (BIS) as covariates that may be associated with which reconstruction options a participant recalls discussing with her surgeon. Univariate multinomial logistic regression model results are presented in Supplemental Table 1. BMI, ASI-R, BIS, and global severity index had type III *p* values less than 0.2. These factors were included in the feature selection process for the multivariable model.

### Multinomial logistic regression

Psychological distress (GSI from BSI-18) was removed from further consideration during backwards model selection on the basis of the Akaike information criterion. The most parsimonious model identified BMI, body image investment (ASI-R), and body image (BIS) as statistically significantly associated with the reconstructive options that a participant remembered discussing with her surgeon. Results from the selected model are summarized in Table 2, with the reference group being participants who remembered discussing only a tissue-based option. The results with each of the other groups taken as the reference are provided in Supplemental Tables 2 and 3. Greater body image investment (i.e., higher ASI-R composite score) was statistically significantly associated with higher odds of remembering discussing only implant-based reconstruction as compared to remembering discussing only tissue-based reconstruction, adjusted for the other factors in the model (adjusted odds ratio [OR] = 1.70, 95% CI 1.04–2.79, *p* = 0.035). Greater body image dissatisfaction or concerns (i.e., higher BIS score) was associated with higher odds of remembering discussing only tissue-based reconstruction compared to remembering discussing only implant-based reconstruction, adjusted for the other factors in the model (adjusted OR = 1.04, 95% CI 1.00–1.08, *p* = 0.045). Note that the significance level of a given covariate in the multinomial logistic regression can be different from what was reported above in the descriptive statistics and univariate analyses sections since the multinomial logistic regression model controls for other variables.

**Table 2:** Multivariable multinomial logistic regression model.

Predictors	OR	CI (95%)	*p* value	Response	Type III *p* value
(Intercept)	23.09	2.32–230.15	**0.007**	Implant	
(Intercept)	21.23	1.18–381.27	**0.038**	All	
Body Image Investment (ASI-R)	1.70	1.04–2.79	**0.035**	Implant	0.04
Body Image Investment (ASI-R)	0.92	0.49–1.72	0.793	All	
Body Mass Index (BMI)	0.86	0.81–0.91	** < 0.001**	Implant	< 0.001
Body Mass Index (BMI)	0.89	0.83–0.96	**0.002**	All	
Body Image Scale (BIS)	0.96	0.92–1.00	**0.045**	Implant	0.1
Body Image Scale (BIS)	0.96	0.92–1.02	0.169	All	
Observations	306				
R^2^ Nagelkerke	0.188				

The reference group was patients who responded that they remembered tissue-based options only. R^2^ Nagelkerke is one type of pseudo R^2^ used as goodness-of-fit measure.

*OR* odds ratio; *CI* confidence interval.

Significant values are in [bold].

## Discussion

Our findings highlight body image investment and body image as important psychological factors that may influence what patients remember from consultations about breast reconstruction. We found that even when adjusted for BMI, patients with greater body image dissatisfaction (BIS) were more likely to remember discussions about tissue-based reconstruction, and patients with higher body image investment (ASI-R) were more likely to remember discussions about implant-based reconstruction.

Patients with cancer are at risk for experiencing body image disturbance due to appearance and functional changes that result from their illness and its treatment. Body image refers to how a person perceives their appearance and body functioning^42^ and can be an important component of well-being more generally, such as in measures of self-compassion^43^ and quality of life^44^. The majority of studies on cancer and body image have focused on breast cancer. Breast cancer treatment has a negative impact on body image^45^, but breast reconstruction offers many women an opportunity to regain a more positive body image^46,47^. Body image investment refers to the degree to which an individual values their appearance and believes their self‐worth is contingent upon appearance. Patients with high levels of body image investment are more likely to direct attention to, encode, and interpret stimuli related to bodily experiences in a negative fashion^48^. Differences in the importance women place on their appearance may help explain differences in adjustment to breast cancer and satisfaction with reconstruction outcomes^34,49–51^. Our study enriches the research on body image concerns of breast cancer patients by demonstrating an association between these concerns and patients’ memories of consultations with their healthcare providers, which are crucial for informed consent and shared decision-making.

Previous research on psychological factors that are associated with what patients remember has focused on measures such as anxiety and depression. For example, during consultation, if a breast cancer patient receives results from a worried physician, they may feel more distress and recall significantly less information^52^. Interestingly, we did not find psychological distress (global severity index from BSI-18, encompassing subscales for depression, anxiety, and somatization) to be significantly associated with the reconstruction options that patients remembered discussing with their surgeon. A plausible explanation is that consultation about breast reconstruction can be one of the more positive experiences of breast cancer care. In previous studies by our team^53^ and others^54^, patients expressed that breast reconstruction gave them a sense of hope and something to look forward to.

We acknowledge that there are some limitations in this study. First, the racial distribution in our sample does not fully reflect the diversity of the United States. Caucasians made up 74.8% (N = 229) of the sample; in contrast, 61.6% of Americans identified as Caucasian only in the 2020 census^55^ and indicates some demographic groups are underrepresented in our sample. Second, the participants in this study reported low levels of distress: the median global severity index score was 4 for participants who remembered discussing all reconstruction categories, 5 for participants who remembered discussing only an implant-based option; and 6 for participants who remembered discussing only a tissue-based option. For reference, the range of the global severity index from the BSI-18 is 0 to 72, with higher scores indicating more psychological distress. Thus, caution is recommended when applying the conclusions of this study to breast cancer patients experiencing higher levels of distress. Lastly, this study purposefully focused on the role of patients’ psychosocial factors that may influence which breast reconstruction options they remember; future studies will explore the effects of provider factors and medium on communication, recall, and comprehension.

We have identified body image and body image investment as new dimensions for future research on patients' ability to accurately recall medical information. Tailored counseling approaches may be recommended to ensure all patients equally attend to and consider all medically-relevant options when discussing breast reconstruction surgery. Moreover, our findings have implications for other diseases, conditions, and treatments that can substantially alter the patient's appearance, such as head and neck cancer.

## Supplementary Information

Supplementary Information.

## Data Availability

The datasets analyzed and presented in this article are available publicly at the Texas Data Repository. The datasets are available at this web address: https://doi.org/10.18738/T8/PLUWD6. Please cite this paper accordingly.

## Code availability

The code generated during the current study is available at https://github.com/UTBiomedicalInformaticsLab/OptionDiscussed_release.git. Please cite this paper accordingly.
