# Supplementary material for: The role of psychosocial factors in patients’ recollections of breast reconstruction options discussed with their surgeons
Source: Sci Rep. 2022 May 6;12:7485. doi: 10.1038/s41598-022-11478-0 (PMC9076612; doi:10.1038/s41598-022-11478-0)
Supplement: Supplementary file 1 — Supplementary Information. [file 41598_2022_11478_MOESM1_ESM.docx]

| Supplement Table 1. Univariate Multinomial Logistic Regression Models* |
| --- |
| \| *Predictors* \| *OR* \| *CI (95%)* \| *p value* \| *Response* \| *Type III p value* \| \| --- \| --- \| --- \| --- \| --- \| --- \| \| Age \| 0.99 \| 0.97 – 1.02 \| 0.654 \| implant \| 0.64 \| \| Age \| 1.01 \| 0.98 – 1.05 \| 0.559 \| all \|  \| \| Body Image Investment (ASI-R) \| 1.49 \| 0.98 – 2.29 \| 0.064 \| implant \| 0.045 \| \| Body Image Investment (ASI-R) \| 0.80 \| 0.45 – 1.41 \| 0.444 \| all \|  \| \| Body Mass Index (BMI) \| 0.84 \| 0.79 – 0.89 \| **<0.001** \| implant \| <0.001 \| \| Body Mass Index (BMI) \| 0.88 \| 0.82 – 0.94 \| **<0.001** \| all \|  \| \| Body Image Scale (BIS) \| 0.95 \| 0.92 – 0.98 \| **0.003** \| implant \| 0.004 \| \| Body Image Scale (BIS) \| 0.94 \| 0.90 – 0.99 \| **0.015** \| all \|  \| \| Global Severity Index (from BSI-18) \| 0.98 \| 0.95 – 1.00 \| 0.098 \| implant \| 0.099 \| \| Global Severity Index (from BSI-18) \| 0.96 \| 0.92 – 1.01 \| 0.082 \| all \|  \| |

*The reference group was patients who responded that they remembered tissue-based options only. R^2^ Nagelkerke is one type of pseudo R^2^ used as goodness-of-fit measure.

Abbreviations: OR, odds ratio; CI, confidence interval

| Supplement Table 2. Multivariable Multinomial Logistic Regression Model* |
| --- |
| \| *Predictors* \| *OR* \| *CI (95%)* \| *p value* \| *Response* \| \| --- \| --- \| --- \| --- \| --- \| \| (Intercept) \| 0.05 \| 0.00 – 0.85 \| **0.038** \| tissue \| \| (Intercept) \| 1.09 \| 0.07 – 18.01 \| 0.953 \| implant \| \| Body Image Investment (ASI-R) \| 1.09 \| 0.58 – 2.03 \| 0.793 \| tissue \| \| Body Image Investment (ASI-R) \| 1.85 \| 1.01 – 3.38 \| **0.045** \| implant \| \| Body Mass Index (BMI) \| 1.12 \| 1.04 – 1.21 \| **0.002** \| tissue \| \| Body Mass Index (BMI) \| 0.96 \| 0.89 – 1.04 \| 0.311 \| implant \| \| Body Image Scale (BIS) \| 1.04 \| 0.98 – 1.09 \| 0.169 \| tissue \| \| Body Image Scale (BIS) \| 1.00 \| 0.95 – 1.05 \| 0.894 \| implant \| \| Observations \| 306 \| \| \| \| \| R^2^ Nagelkerke \| 0.188 \| \| \| \| |

*The reference group was patients who responded that they remembered all options. R^2^ Nagelkerke is one type of pseudo R^2^ used as goodness-of-fit measure.

Abbreviations: OR, odds ratio; CI, confidence interval

| Supplement Table 3. Multivariable Multinomial Logistic Regression Model* |
| --- |
| \| *Predictors* \| *OR* \| *CI (95%)* \| *p value* \| *Response* \| \| --- \| --- \| --- \| --- \| --- \| \| (Intercept) \| 0.04 \| 0.00 – 0.43 \| **0.007** \| tissue \| \| (Intercept) \| 0.92 \| 0.06 – 15.23 \| 0.953 \| all \| \| Body Image Investment (ASI-R) \| 0.59 \| 0.36 – 0.96 \| **0.035** \| tissue \| \| Body Image Investment (ASI-R) \| 0.54 \| 0.30 – 0.99 \| **0.045** \| all \| \| Body Mass Index (BMI) \| 1.17 \| 1.10 – 1.24 \| **<0.001** \| tissue \| \| Body Mass Index (BMI) \| 1.04 \| 0.96 – 1.12 \| 0.311 \| all \| \| Body Image Scale (BIS) \| 1.04 \| 1.00 – 1.08 \| **0.045** \| tissue \| \| Body Image Scale (BIS) \| 1.00 \| 0.95 – 1.06 \| 0.894 \| all \| \| Observations \| 306 \| \| \| \| \| R^2^ Nagelkerke \| 0.188 \| \| \| \| |

*The reference group was patients who responded that they remembered implant-based options only. R^2^ Nagelkerke is one type of pseudo R^2^ used as goodness-of-fit measure.

Abbreviations: OR, odds ratio; CI, confidence interval
